# Titration methods for rVSV-based vaccine manufacturing

**DOI:** 10.1016/j.mex.2020.100806

**Published:** 2020-02-20

**Authors:** Jean-François Gélinas, Sascha Kiesslich, Rénald Gilbert, Amine A. Kamen

**Affiliations:** a Department of Bioengineering, McGill University, 3480 University, Montréal, Québec H3A 0E9, Canada; b Human Health Therapeutics, National Research Council Canada, Montreal, QC, Canada

**Keywords:** rVSV-ZEBOV, Ebola, rVSV-HIV, HIV, dPCR, TCID_50_

## Abstract

The recombinant Vesicular Stomatitis Virus (rVSV) is an emerging platform for viral vector-based vaccines. Promising results have been reported in clinical trials for the rVSV-ZEBOV vaccine for Ebola virus disease prevention. In this study, we describe the titration tools elaborated to assess the titre of rVSV-ZEBOV productions.

• A streamlined Median Tissue Culture Infectious Dose (TCID_50_) assay to determine the infectious titer of this vaccine was established.

• A digital polymerase chain reaction (dPCR) assay to assess the total number of viral particles present in cell-free culture supernatants of rVSV productions was developed.

• These assays are used to titre rVSV-ZEBOV samples and characterize the ratio of total particles to infectious units for monitoring process robustness and product quality attributes and can be used to titre samples generated in the production of further rVSV vectors.

**Table:** Specifications table

Subject Area	*Biochemistry, Genetics and Molecular Biology*
More specific subject area:	*Virus titration*
Method name:	*TCID_50_ and dPCR*
Name and reference of original method	Reed LJ, Muench H. A Simple Method of Estimating Fifty Per Cent Endpoints12. American Journal of Epidemiology. 1938;27:493-7.

## Background

The recombinant Vesicular Stomatitis Virus (rVSV) platform is a replication-competent vaccine that has been shown to generate both cell-mediated and humoral immunity to expressed foreign antigens [1] and is being developed to target several infectious diseases and cancers [2]. One of these targets, the Ebola virus disease, is an urgent international priority, hence there is intensive research activity surrounding the development of a safe and efficacious vaccine. Recently, the Ebola virus vaccine Ervebo has been approved by the FDA [3], prequalified by the World Health Organization and granted a conditional marketing by the European Commission [4]. This vaccine is based on the rVSV-ZEBOV, an attenuated, replication-competent rVSV pseudotyped with the Ebola Zaire glycoprotein [5].

Replication-competent vaccine titres can be quantified by a functional titre, an enumeration of the infectious virus particles, and/or by a particle titre, an enumeration of the number of particles present in a volume of vaccine. The combination of a functional assay with a total particle assay through their ratio (functional particles over total particles) is a critical quality attribute for the characterization of the candidate rVSV-based vaccine as defective interfering rVSV particles have been shown to modulate virulence [6].

The endpoint dilution assay (TCID_50_) [7] is a measure of the functional titre of samples as it quantifies the number of transducing particles required to produce a cytopathic effect in 50% of inoculated tissue culture cells. TCID_50_ and plaque-forming units (pfu) are reliable techniques and routinely used to titre vaccine preparations [8].

The total particle count is usually established using quantitative polymerase chain reaction (qPCR). Specific fluorescent labeling is used to measure the progress of PCR in real time and allows for quantification of the DNA template. A standard curve generated from a serial dilution of DNA standard of known concentration is used to evaluate the qPCR result. In contrast, the digital PCR (dPCR) method is a relatively novel tool that can be used to obtain a total particle count without the need for a standard curve [9,10]. Here, around 20,000 water-oil emulsion droplets are generated from one sample and during the PCR step, specific DNA amplification is carried out within each droplet. The method takes advantage of this template DNA separation to partition the sample and to enable individual droplet analysis following the PCR. By counting positive and negative reactions, the method determines the value of viral genomes per mL. Because of this absolute quantification, no standard curve is required, and the workflow is simplified [11]. Recently, dPCR has been directly compared to qPCR indicating higher precision and reproducibility in the case of dPCR [12]. Further, dPCR has shown better intra- and inter-assay precision than qPCR without the issues related to the use of plasmids as DNA reference material in the case of quantification of the number of total genomes for rAAV vectors [13,14].

Here we describe a TCID_50_ method as well as a dPCR method for titration of rVSV samples. Certain aspects that can affect the variability of these assays are explored and the dPCR method is tested against three different variants of rVSV vectors.

## Method details

### Cells

HEK 293A cells [15] (American Type Culture Collection, Manassas, VA, USA) were maintained in cell culture dishes (Greiner Bio-One, Kremsmünster, Austria), in a humidified incubator (Thermo Fisher Scientific, Waltham, MA, USA) at 5% CO_2_ and 37 °C in Dulbecco’s Modified Eagle’s Medium (DMEM) (Thermo Fisher Scientific), supplemented with 2 mM L-glutamine and 5% Fetal Bovine Serum (FBS) (GE Healthcare) without antibiotics. Cells were passaged twice a week. The confluent cells were detached using a cell scraper, centrifuged at 500 × g for 5 min, resuspended in fresh medium and seeded at a 1:10 dilution.

### Viruses

The generation of rVSV-EboGP B6 (rVSV-ZEBOV) [16,17] and rVSV-GFP [18] samples has been described previously. Sample generation for rVSV-EboGP B6-NL4.3Env/SIVtm (rVSV-HIV) will be described in detail in upcoming publications. Briefly, the genome plasmid used for the production of rVSV-ZEBOV was modified to include the Env sequence of NL4.3 with an SIV transmembrane domain. The generation of the lentiviral vector (LV) used to validate the rVSV primers has been described previously [19].

### Median tissue culture infectious dose (TCID_50_)

The endpoint dilution assay [20] was used as a measure of the functional titre of virus samples as it quantifies the number of transducing particles required to produce a cytopathic effect in 50% of inoculated tissue culture cells. rVSV titration on HEK 293A was previously shown to give similar results as titration on Vero cells [18]. HEK 293A cells were seeded at ~5 × 10^4^ live cells per mL in 96 well plates (Greiner Bio-One, Kremsmünster, Austria), referred to as the TCID_50_ plates, with 100 µL per well one day before initiating the titration. Virus dilution series of twelve times 1:5 were prepared in separate 96 V bottom well plates (Sarstedt, Nümbrecht, Germany), referred to as the dilution plates, using 50 µL in 200 µL of HyClone HyCell TransFx H. Eight dilution series were performed per dilution plate. Using a multichannel pipette, 20 µL per well of each individual dilution series were transferred into each of the eight rows of a single TCID_50_ plate. The TCID_50_ plates were incubated at 37 °C and 5% CO_2_. Cytopathic effect was observed after at least 7 days incubation and scored by standard light microscopy. The TCID_50_/mL value was calculated by the Spearman & Kärber algorithm [21,22] as described previously [23].

### Digital polymerase chain reaction (dPCR)

To estimate the total number of viral particles present in cell-free culture supernatants, the copy number of viral genomes was assessed by dPCR. Viral RNA was extracted using the High Pure Viral Nucleic Acid Kit (Roche, Mannheim, Germany) according to the manufacturer's instructions. The input-volume was kept at 200 µL for all samples, and purified RNA was eluted into 50 µL elution buffer. This kit has been shown to have better recovery rate than others in a side-by-side comparison of five different nucleic acid extraction kits on a different DNA virus (Hepatitis B virus) possibly because of the use of a proteolytic enzyme in addition to the chemical denaturants [24]. The viral RNA was then reverse transcribed using the iScript Select cDNA synthesis kit (Bio-Rad Laboratories) according to the manufacturer's instructions and using gene-specific primers targeted towards the viral L-protein (polymerase) amplifying a 114 base pair fragment. The primer sequence for the forward primer was [5′- CTGCTGTCCGGAATCAGGTT-3′] and for the reverse primer [5′- GCCGTCTCCACAACTCAAGA-3′] (Integrated DNA Technologies, Inc., Coralville, IA, USA).The cDNA reaction mix consisted of 4 µL of 5x iScript reaction mix, 0.5 µL of each primer at a concentration of 10 µM, 2 µL of GSP enhancer solution, 2 µL of purified RNA from the extraction, 1 µL of reverse transcriptase, and 10 µL of purified and nuclease-free water. The dPCR was performed using the QX200™ Droplet Digital™ PCR System with the EvaGreen Supermix (Bio-Rad Laboratories) following the manufacturer's instructions. Briefly, the dPCR reaction mix consisted of 10 µL EvaGreen Supermix, 0.5 µL of each primer at a concentration of 10 µM, 5 µL of cDNA from the reverse transcription step, and 4 µL of purified and nuclease-free water. Using the droplet generator and the corresponding cartridges, the 20 µL reaction mix was combined with 65 µL of EvaGreen droplet generation oil to generate droplets. The droplets were then transferred to dPCR 96-well plates where the dPCR reaction took place. Thermocycling conditions were as follows: initial denaturation at 95 °C for 5 min followed by 35 cycles at 95 °C for 30 s, 57 °C for 60 s, and 72 °C for 30 s, followed by a final extension at 72 °C for 5 min. The annealing temperature of 57 °C was established in a preliminary temperature gradient experiment. An internal virus reference standard, consisting of a new vial taken from a viral seed stock generated as described previously [16], was run in every assay alongside to ensure repeatability of the whole assay including RNA extraction. In addition, a non-template control was included in each run to rule out the contamination of the PCR reagents. The analysis of the droplet read after dPCR-amplification resulted in a value which, after correction for the dilution factor, gave the number of copies of viral genomes, which contain the selected sequence, present in the original sample with the unit of VG/mL.

To demonstrate the trueness of the assay, the plasmid pATX.V2.Full, which was used to generate the rVSV-ZEBOV [16], was analyzed by ddPCR using the protocol above. The resulting gene copy number was compared to the gene copy number determined via spectrophotometry. The amount of DNA in the plasmid sample was determined using a ND-2000 spectrophotometer (NanoDrop, Thermo Fisher Scientific). The plasmid copy number was then calculated using the formula:

$$number\;of\;copies\;\left(molecules\right)=\frac{X\;ng\times \;6.022\;\times 10^{23}\;\frac{molecules}{mol}}{N\times 10^{9}\frac{ng}{g}\times \;650\;\frac{g}{mol}}$$

where *X* = amount of DNA in the plasmid sample, *N* = length of the pATX.V2.B6.Full plasmid (14,086 base pairs), and 650 g/mol = average mass per base pairs.

To show specificity of the dPCR method towards rVSV, a lentiviral vector (LV) was titrated using the method developed for rVSV. Viral RNA of the LV was extracted using the same method as for rVSV while reverse transcription was performed using a random primer mix supplied with the cDNA kit. dPCR was performed in triplicates using the same cDNA. The LV-specific primer sequences for the dPCR were: forward primer [5′- GTCCTTTCCATGGCTGCTC -3′] and reverse primer [5′- GCCGTCTCCACAACTCAAGA-3′] (Integrated DNA Technologies).

### Statistical analysis

Mean and standard deviations, represented as error bars in figures, as well as unpaired Welch's t-tests were calculated using Prism 8.2.0 (GraphPad, La Jolla, CA, USA). Statistical power was evaluated using the G*Power 3.1.9.2 software (University of Düsseldorf, Düsseldorf, Germany) [25] using an *a priori* t test to determine the required sample size to observe the difference between two independent means with an α error probability of 0.05.

## Results

### Variability of titration using TCID_50_

**Fig. 1:**
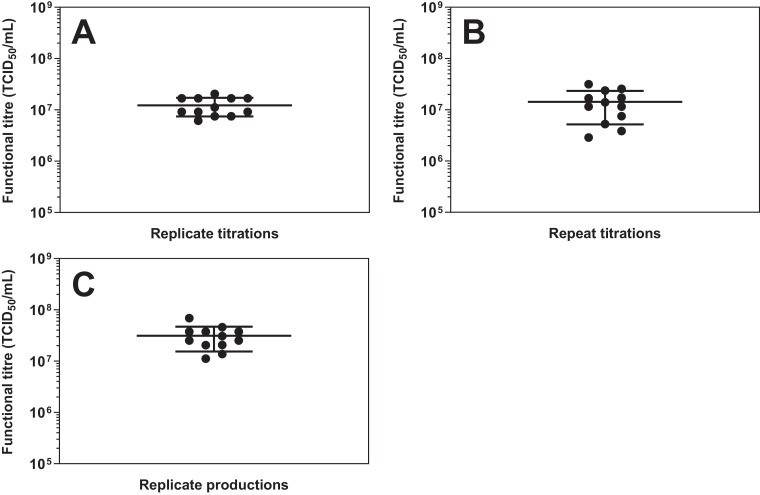
Production and titration variability using TCID_50_. Functional titres were measured by TCID_50_. Bars represent the mean of the twelve samples ± standard deviation. A) Titration repeatability. Independent titration by TCID_50_ in 12 replicates on the same day of a single production sample. B) Titration intermediate precision. Independent titrations by TCID_50_ repeated on 12 separate days for aliquots of the same production. C) Production repeatability. Production yields for 12 independent infections with rVSV-ZEBOV at MOI 0.001 of two 6 well plates containing 1 × 10^6^ cells/mL in 2 mL per well.

To evaluate the repeatability of rVSV-ZEBOV titration using this method, twelve parallel TCID_50_ evaluations of a single sample of an rVSV-ZEBOV seed stock were performed using the same procedure, operator, measuring system, operating conditions and location. The functional titre of that production batch was evaluated to be 1.23 ×  10^7^ TCID_50_/mL (standard deviation: 4.88 × 10^6^) (Fig. 1A). The intermediate precision was also assessed by titration of the same sample on twelve different days over months (Fig. 1B). As expected, intermediate precision was shown to be more variable than the assay's repeatability with an average titre of 1.43 ×  10^7^ TCID_50_/mL and a standard deviation of 9.10 × 10^6^. When performing an unpaired Welch's test between the results from the replicate titrations and the repeat titrations, a significant difference (p<0.0499) was found between the variances. The intermediate precision of this assay has been reported before in the titration of filovirus where the range was approximately 1.5 log [26]. Hence, each of the samples presented in the same figure should be titrated on the same day to avoid the added impact of interday variability. To further reduce variability, an automated process could be developed and would limit operator variability.

The repeatability of virus production was also assessed to determine its impact on the evaluation of the titre of a sample as well as the number of replicates necessary to have sufficient power to observe statistical significance in production experiments where different parameters are evaluated. The production of rVSV-ZEBOV was evaluated in multiple independent infections using two 6 well plates seeded with HEK 293SF cells and again using the same procedure, operator, measuring system, operating conditions and location. These were infected with rVSV-ZEBOV at a multiplicity of infection (MOI) of 0.001 and left to incubate with agitation for 2 days at 34 °C. The functional viral titre for each of the 12 independent cultures, as determined by TCID_50_, is shown in Fig. 1C (mean of twelve wells: 3.13 × 10^7^ TCID_50_/mL, standard deviation: 1.59 × 10^7^). Using these data, to model future studies incorporating three replicates, statistical power analysis demonstrated that a minimum of a 2.26-fold increase in functional titre would be necessary to observe a statistical difference with 80% power using triplicates and accounting for the variability of the TCID_50_ assay if performed for all samples on the same day.

### Variability of titration using dPCR

**Fig. 2:**
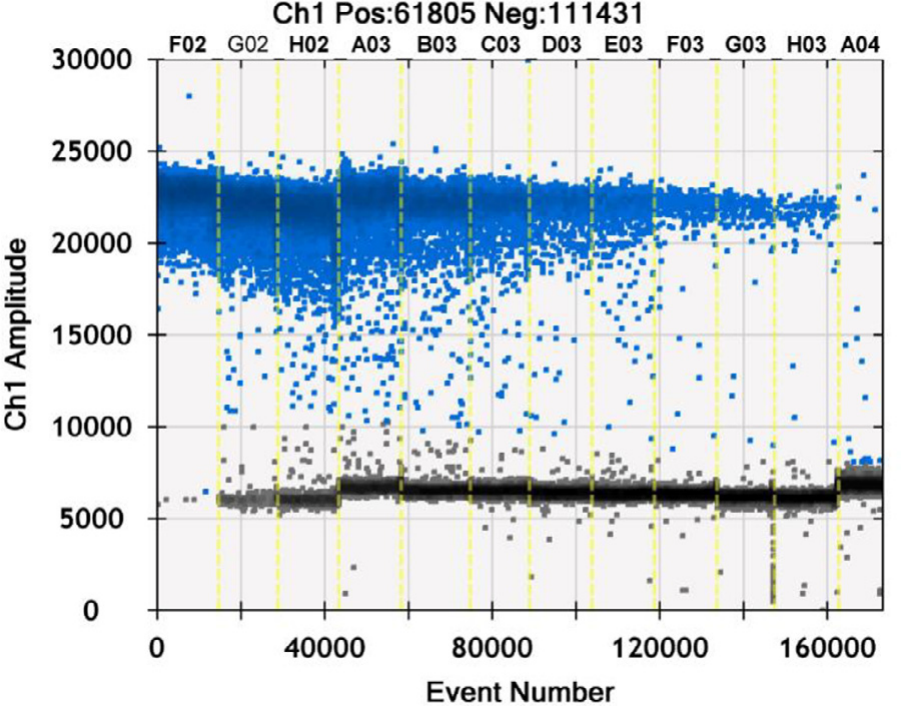
Histogram of dPCR analysis of a dilution series of cDNA extracted from rVSV-ZEBOV. The extracted cDNA was diluted in a 1:2 dilution series starting from 1:100 (sample F02) to 1:102,400 sample (H03). Sample A04 consisted of a non-template control. Channel 1 amplitude is given in arbitrary fluorescent units. Positive events are marked in blue, negative events in gray.

**Fig. 3:**
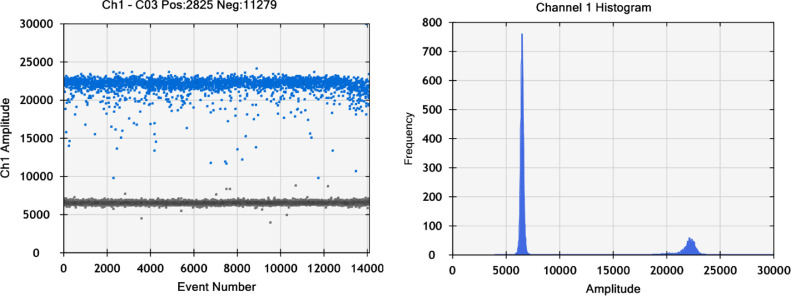
Histogram of dPCR analysis of rVSV-ZEBOV. This exemplary histogram shows the data of an rVSV-ZEBOV sample at a cDNA dilution resulting in a clear peak resolution of dPCR events. Channel 1 amplitude is given in arbitrary fluorescent units. Positive events (blue) typically peaked around 20,000 to 25,000 and negative events (gray) peaked between 5000 and 8000.

For any given sample to be analyzed by dPCR, the cDNA needs to be diluted appropriately prior to the run for the purpose of achieving clear peak resolution of dPCR events and so that the resulting dPCR signal falls within the linear dynamic range of analysis for accurate measurements according to the manufacturer's instructions. A histogram of the dPCR analysis of a dilution series of a cDNA sample extracted from rVSV-ZEBOV is shown in Fig. 2. As expected, the least diluted samples showed almost only positive events due to the abundance of gene copies. The more the sample got diluted, the less positive and the more negative events occurred. The most diluted sample showed only a few positive events and mostly negative events. The histogram from the sample with 1:3200 dilution (sample C03) is individually shown in Fig. 3 and shows a clear peak resolution of dPCR events without significant amount of rain. Positive events typically peaked at around 20,000 to 25,000, whereas negative events peaked between 5000 and 8000.

To demonstrate that the developed dPCR method is measuring the correct amount of gene copies, a plasmid containing the VSV polymerase gene sequence was analyzed via dPCR and the gene copy number was verified via spectrophotometry. The amount of DNA in the plasmid sample was measured by spectrophotometry and was 371 ng/µL. Using the formula stated in the methods, the resulting number of gene copies was determined to be 2.44 × 10^13^ per mL. In comparison, the number of gene copies determined via dPCR was 3.48 × 10^13^ per mL, indicating an appropriate correlation between the two methods.

**Fig. 4:**
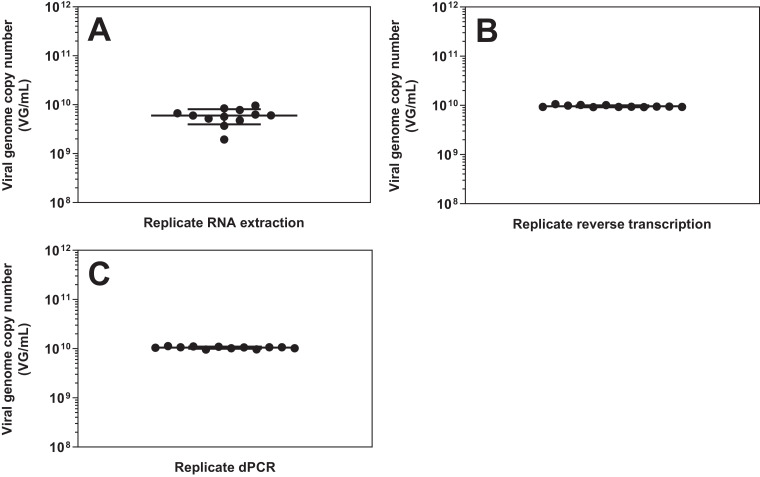
Titration variability of rVSV-ZEBOV using dPCR. Viral genome copy number was measured by dPCR. Bars represent the mean of the twelve samples ± standard deviation. A) Total assay variability. dPCR analysis of the same sample with 12 independent RNA extractions. B) Combined reverse transcription and dPCR variability. dPCR analysis of the same RNA extract with 12 independent reverse transcriptions. C) dPCR variability. dPCR analysis was performed 12 times of the same cDNA.

To estimate the repeatability of the dPCR assay, the number of viral genomes in the rVSV-ZEBOV seed stock was quantified in 3 different approaches. To evaluate the total variability of the assay, twelve separate RNA extractions were performed on the same sample. Each extraction was then reverse transcribed into cDNA and analyzed by dPCR. The average titre was 6.01 × 10^9^ VG/mL (standard deviation: 2.06 × 10^9^) (Fig. 4A).

To evaluate which step led to the most variability, one RNA extraction sample from the previous experiment was used and, reverse transcription was carried out 12 times resulting in 12 separate cDNA batches. Each of these cDNA batches was analyzed individually by dPCR. Here, the average titre was 9.61 × 10^9^ VG/mL (standard deviation: 4.62 × 10^8^) (Fig. 4B). When performing an unpaired Welch's test between the results from the replicate RNA extraction and the replicate reverse transcription, a significant difference (p<0.0001) was found between the variances, indicating that the RNA extraction step leads to a significantly greater variance compared to the RNA transcription step with regards to the final dPCR titer. Finally, from one of the cDNA batches, twelve dPCR were performed separately. The average titre was 1.05 × 10^10^ VG/mL (standard deviation: 5.23 × 10^8^) (Fig. 4C). An unpaired Welch's test between the results from the replicate reverse transcription and the replicate dPCR from the same cDNA found no significant difference between the variances (p=0.6884). Hence, the main source of error of the dPCR assay was found to be the step of viral RNA extraction. Whereas reverse transcription and dPCR are one step reactions, the RNA extraction involved multiple steps including viral particle disruption, RNA binding to the membrane, inhibitor removal, washing and elution of the extracted RNA. Besides, viral RNA is relatively unstable compared to DNA.

### Titration using dPCR for other strains of rVSV

In addition to the titration of rVSV-ZEBOV, the method was further tested by titrating two other significant rVSV strains: rVSV-GFP and rVSV-HIV. rVSV-HIV is currently being studied as a promising vaccine candidate against human immunodeficiency virus infection where the glycoprotein of VSV has been replaced by HIV's Env glycoprotein [27]. rVSV-GFP is taking advantage of GFP expression to study, for example, the viral life cycle, but still expresses the wild-type glycoprotein (VSV-G) [18,28]. TCID_50_ was performed in triplicate on a single rVSV-HIV and rVSV-GFP sample. The functional titres of these samples were 8.07 × 10^7^ (standard deviation: 3.78 × 10^7^) and 4.03 × 10^9^ (standard deviation: 1.28 × 10^9^) respectively. Viral RNA from rVSV-HIV and rVSV-GFP was extracted and reverse transcribed as before. In both cases, the dPCR was performed in triplicates using the same cDNA. The genomic titre of the sample of rVSV-HIV was determined to be 2.67 × 10^10^ VG/mL (standard deviation: 3.06 × 10^8^) and 1.86 × 10^10^ VG/mL (standard deviation: 9.24 × 10^8^) for the rVSV-GFP sample.

To show specificity of the dPCR method towards rVSV, a lentiviral vector (LV) was titrated using the method developed for rVSV. LV showed no PCR amplification when using rVSV-specific primers for the dPCR step. As a control, LV showed PCR amplification when using LV-specific primers. In contrast, cDNA of rVSV-ZEBOV generated as in Fig. 2 did not show dPCR amplification using these LV-specific primers.

## Conclusion

In this work, we described an assay to determine the functional titre of rVSV vectors (TCID_50_) as well as an assay to determine the number of viral genomes (dPCR). The later can be used to estimate the total number of viral particles. Together, these assays provide reliable methods to determine significant values in viral vaccine bioprocess development.

Depending on the rVSV strain, the TCID_50_ assay can require up to seven days before reading of the cytopathic effect. Immunoperoxidase staining could help improve and speed up the process of distinguishing positive and negative wells. It is however important to note that, because of a strong cytopathic effect in the case of rVSV-ZEBOV, positive wells show no surviving cells after a few days while in negative wells cells are completely intact. An alternative method to quantify the infectious viral titer of VSV has been recently published which uses laser force cytology [29]. This method has the advantage of reducing the time required until the titer can be determined. It still needs to be demonstrated that this method can be applied to other recombinant strains of VSV with similar reduction in assay time. However, the benefit of the TCID_50_ assay is its simplicity which can be carried out in standard equipped laboratories without the need for complex instruments.

In contrast, the dPCR assay can be completed within one day and can, therefore, be used as a preliminary estimation of the viral titre if necessary. Nevertheless, like other vectors, rVSV typically produces a variety defective interfering particles during infection [30,31]. In this study, only one methodology was used to determine each, the infectious and the total particle count, respectively, to get analytical data that can be used in the development of the upstream bioprocess. To more accurately determine the number and molecular diversity of the defective interfering particles that are created during the rVSV-ZEBOV vaccine production process, further analytical methods would be required. This would allow for additional insights into the biological complexity of rVSV production, further enabling bioprocess improvements. For example, the dPCR method developed in this work targets only those particles which contain the sequence of the VSV polymerase gene. The method does not consider defective particles lacking this gene sequence. A physicochemical approach to determine the number of total particles, for example via HPLC, could be a valuable extension of the analytical methods tool box for rVSV total viral particle quantification.

Despite these limitations, the developed method to estimate the number of total particles can still generate very useful data. For example, following the production step, dPCR would allow the confirmation of a successful production of vectors as well as the number of total viral particles which is useful to proceed with the downstream processing of viral vectors with an appropriate concentration step. Since both the number of infectious particles and total particles are expected to change throughout the purification process, the dPCR assay can be a sufficient quantification method enabling continuation to the next step with the benefit of reducing the required time for process analytics in between the upstream and downstream steps. We further demonstrated that these assays are applicable to rVSV-ZEBOV, a very relevant candidate vaccine and that the dPCR can be used for other recombinant strains of VSV. Different pseudotyped rVSV are currently being investigated as vaccine candidates, and further, as possible oncolytic viruses. In particular, rVSV-HIV and rVSV-GFP were successfully titrated using the same dPCR method as used for rVSV-ZEBOV. Since the primers used in the dPCR assay are targeting a sequence of the VSV polymerase (L-gene), this assay can serve as a universal method to determine the number of viral genomes of other rVSV as long as the L-protein sequence remains wild-type. It has been further demonstrated that the developed dPCR method is specific for rVSV vectors and does not result in dPCR amplification of other viral vectors.

## Appendix Supplementary materials

Image, application 1
